# Clinical Implications and Risk Factors for Erectile Dysfunction in Kidney Transplant: A Single-Center Assessment

**DOI:** 10.7759/cureus.38088

**Published:** 2023-04-24

**Authors:** Adelina Miron, Ionut Nistor, Corneliu Morosanu, Lucian Siriteanu, Adrian Covic

**Affiliations:** 1 Department of Urology, “Grigore T. Popa” University of Medicine and Pharmacy, Iasi, ROU; 2 Department of Urology and Renal Transplantation, “Dr. C.I. Parhon” Hospital, Iasi, ROU; 3 Department of Nephrology, “Grigore T. Popa” University of Medicine and Pharmacy, Iasi, ROU; 4 Department of Nephrology, “Dr. C.I. Parhon” Hospital, Iasi, ROU; 5 Department of Vascular Surgery, “Grigore T. Popa” University of Medicine and Pharmacy, Iasi, ROU

**Keywords:** sexual dysfunction, kidney transplant recipients, chronic kidney disease, erectile dysfunction, renal transplant

## Abstract

Background: Erectile dysfunction (ED) affects the great majority of people undergoing dialysis and also the majority of patients undergoing kidney transplantation. In this study, we investigated the degree of erectile dysfunction (ED), as well as its prevalence, contributory variables, and overall impact after renal transplant.

Methods: Adult male kidney transplant patients were the subject of an observational, non-interventional study that was conducted at a single center. Age, time and type of dialysis before transplantation, comorbidities, factors associated with cardiovascular risk, data on sexual history, physical examination, and laboratory results were among the clinical data we examined. In addition to gathering clinical and demographic characteristics, the International Index of Erectile Function (IIEF) questionnaire was used to evaluate sexual function.

Results: A total of 170 renal transplanted patients between 20 and 70 years old (mean age: 45.40±11.5) were included in this study. All of the patients had immunosuppressive treatment with a calcineurin inhibitor (cyclosporine or tacrolimus) and had a normal glomerular filtration rate (GFR). The prevalence of sexual dysfunction increased with age (42.6% of patients under 40, 47.4% of patients in the 40-60 age group, and 78.9% of patients over 60). Mild, moderate, and severe ED was noted in 33.5%, 20.6%, and 10.6% of cases, respectively, and 51 (30%) patients reported having a normal sexual function. While calcium channel blockers (122 cases) were the most commonly used antihypertensive medication and chronic glomerulosclerosis (55.3%) was the most common cause of chronic kidney disease (CKD) before transplantation, none of these variables appear to have affected the severity of erectile dysfunction. The only medications associated with sexual dysfunction were alpha-blockers and aspirin (75 mg) (p=0.026 and p=0.013, respectively).

Conclusions: Although kidney transplantation has positive impacts on the quality of life, erectile dysfunction is a frequent condition among patients with renal transplants, and it has an increased frequency with age. In our study, it has been observed that only a small percentage of the research group had a normal sexual function, although most of the patients were young, and that alpha-blockers and aspirin (75 mg) are associated with erectile dysfunction.

## Introduction

Chronic kidney disease (CKD) considerably burdens a person’s quality of life. Several comorbidities, including diabetes, hypertension with secondary cardiovascular comorbidities, metabolic syndrome, endocrinological disorders, cognitive and psychiatric diseases, and a prolonged uremic environment, are related to CKD [1]. These diseases and the medications used to treat them may affect erectile dysfunction (ED), a crucial aspect of life quality from both a psychological and a social point of view. Although the quality of life following a kidney transplant improves, the statistics on patients’ sexual behavior have produced contradictory outcomes up to this point. The risk factors for erectile dysfunction (ED) are similar to those for chronic kidney disease (CKD), which can include vascular, neurological, hormonal, or psychological factors. Growing evidence demonstrates that renal failure is associated with sexual dysfunction, anxiety, depression, and a poor quality of life [2].

Some research has demonstrated that erectile dysfunction improves after renal transplant surgery [3,4], while others have not observed any significant improvements or even appearance of new cases of ED [5,6]. Together with recognized vascular, neurogenic, and psychological aspects, adverse drug reactions such as hypogonadism connected to immunosuppressive steroid usage and interference with penile vascularization are having a significant negative impact on erectile performance [5].

Quality of life, including erectile function, became a significant aspect for both patients and doctors in the modern era of healthcare as graft survival and patient survival with graft increased. There is a lack of data regarding sexual function in transplant recipients; consequently, additional information is required to determine the prevalence, risk factors, and severity of the association between post-kidney transplantation status and erectile dysfunction. The aim of our research was to assess the prevalence of erectile dysfunction among patients with kidney transplants and the frequency of risk factors associated with sexual dysfunction.

## Materials and methods

We carried out a retrospective observational research study on male adult patients with chronic kidney disease who underwent a kidney transplant between 1995 and 2021 and were being monitored at the renal transplant unit of “Dr. C.I. Parhon” Hospital in Iaşi. All of the participants provided written consent, and the hospital’s ethics committee authorized the study. Surgical history, the presence of comorbidities, the type of renal impairment, the initial cause of kidney failure, and demographic and clinical data including blood and urine laboratory tests were gathered. In terms of laboratory results, data were gathered and compared for urea (mg/dL), creatinine (mg/dL), hemoglobin (g/dL), hematocrit (%), cholesterol (mg/dL), triglycerides (mg/dL), and uric acid (mg/dL) at the first evaluation following hospital discharge, the evaluation after 12 months, and at the time of study inclusion. All of these data were collected at the start of 2022.

The International Index for Erectile Function (IIEF), a validated assessment of sexual function, was given to patients. To determine the diagnosis of erectile dysfunction, the questionnaire was completed by the patients at the time of study inclusion, and severity scores were established together with additional patient data. Individuals with acute or chronic graft rejection and patients with genital abnormalities incompatible with sexual activity were excluded from the study. Patients who underwent surgery less than six months before the study began were also excluded, as were individuals who refused to fill out the questionnaire.

The diagnostic criteria established by Rosen et al. [7] and Cappelleri et al. [8] were used to diagnose and assess the severity of ED: severe ED (5-7 points), moderate ED (8-11 points), mild-to-moderate ED (12-16 points), mild ED (17-21 points), and no ED (22-25 points).

Data on patients and completed questionnaires were processed using the Statistical Package for the Social Sciences (SPSS) version 27.0 (IBM SPSS Statistics, Armonk, NY, USA). Student’s t-test was used for the statistical examination of the means and continuous variables when the data were regularly distributed. Statistically significant differences were those with p-values < 0.05.

## Results

The study included 170 adult male patients who fulfilled the inclusion requirements, 20-70 years old (mean age: 45.40±11.5). They responded to the IIEF questionnaire at the start of 2022, having received a kidney transplant between 1995 and 2021.

The clinical details of these 170 patients are shown in Table 1. Nineteen (11.2%) patients were beyond the age of 60, 54 (31.7%) were under the age of 40, and 97 (57.1%) were in the 40-60 age range. In terms of sexual activity, the majority of patients (n=114) had long-term partners. Patients varied from morbidly obese (22.3%) to normal weight (39.4%) based on their biometric data in relation to standard body mass index (BMI) (lowest BMI: 16.98 kg/m^2^, highest BMI: 37.44 kg/m^2^). The majority of the recipients (83.5%) underwent dialysis before the renal transplant; 124 patients had hemodialysis and 18 had peritoneal dialysis. The lengths of renal impairment by dialysis ranged from one month to 25 years (mean: 3.36±4.66 years); 19 patients had been dialyzed for more than 10 years until the kidney transplantation, while 53 patients had been on dialysis for less than 12 months. Regarding the donors, there were either live donors (89 total, of whom 45.9% were related to the patients and 6.5% were not) or cadaveric donors (81 cases, 47.6%). Most cases (70.58%) had been performed 1-10 years before the research, while six patients (3.35%) had their renal transplants more than 20 years before the study. Before they entered the study, 46 (27.1%) patients had undergone one or more reversible rejection events, and 17 (10%) patients had graft biopsies.

**Table 1:** Patient demographic, clinical characteristics, transplant data, and treatment BMI: body mass index

	Characteristics	Overall (N=170)	Percentage
Age on enrolment	≤40 years	54	31.8
	40-60 years	97	57.1
	≥60 years	19	11.2
Age at transplant	≤40 years	101	59.4
	40-60 years	65	38.2
	≥60 years	4	2.4
Alcohol	Yes	17	10
	No	153	90
Smoking	Yes	32	18.8
	No	138	81.2
BMI	Normal	67	39.4
	Overweight	65	38.3
	Obesity	38	22.3
Partner (stable relationship)	Yes	114	67.1
	No	9	5.3
	Not specified	47	27.6
Type of dialysis	Hemodialysis	124	72.9
	Peritoneal dialysis	18	10.6
	Pre-emptive transplant	28	16.5
Time on dialysis	>10 years	19	11.1
	<10 years	120	70.6
	Pre-emptive transplant	31	18.3
Type of donor	Living donor (related)	78	45.9
	Living donor (unrelated)	11	6.5
	Deceased donor	81	47.6
Time since renal transplant	>10 years	50	29.4
	≤10 years	120	70.6
Hypertension	Yes	146	85.9
	No	24	14.1
Diabetes	Yes	16	9.4
	No	154	90.6
Neurological disease	Yes	6	3.6
	No	162	96.4
Urological disease	Yes	31	18.2
	No	139	81.8
Organ rejection	Yes	46	27.1
	No	124	72.9
Surgical complications	Yes	14	8.2
	No	156	91.8
Immunosuppressive therapy	Tacrolimus	113	66.5
	Cyclosporin	57	33.5

**Figure 1:**
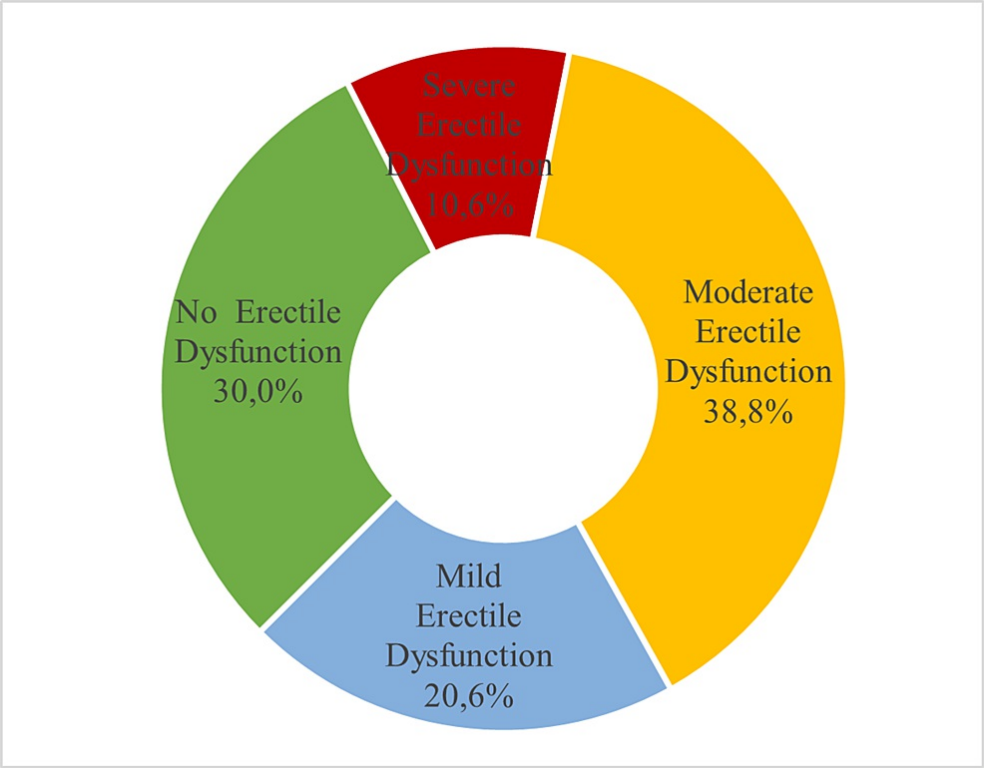
Erectile dysfunction distribution as measured using the International Index for Erectile Function (N=170)

The distribution of erectile dysfunction severity levels is shown in Figure 1: 51 (30%) individuals reported having a normal sexual function, and 38.8%, 20.6%, and 10.6% of participants had moderate, mild, or severe ED, respectively. The mean IIEF score was 16.32±6.93, while the mean scores for the erectile domains were 19.22±7.9 for erectile function, 6.8±2.9 for orgasmic function, 6.43±2.1 for sexual desire, 8.96±3.7 for intercourse satisfaction, and 6.78±2.6 for overall satisfaction. Age-related increases in the prevalence of ED were 42.6%, 47.4%, and 78.9% for patients who were under 40 years old, 40-60 years old, and above 60 years old, respectively.

When looking at the cause of chronic kidney disease before transplantation, chronic glomerulosclerosis (55.3%) was the most frequent cause of chronic kidney disease, followed by hypertensive nephropathy (9.4%), autosomal dominant polycystic kidney disease (ADPK) (7.6%), and urological congenital abnormalities (6.5%), vasculitis (3.9%), and diabetic nephropathy (2.4%).

In addition to taking immunosuppressive medications, the majority of patients (n=159, 93.5%) also received different therapy for comorbidities. Calcium channel blockers (122 cases), beta-blockers (36 cases), and angiotensin-converting enzyme inhibitors (31 cases) were the most frequently used antihypertensives. Alpha-blockers and aspirin (75 mg) (p=0.026 and p=0.013, respectively) were shown to be statistically significantly related to the existence of sexual dysfunction, not only with the overall IIEF score but also with each of the five domains. The associations between medication and erectile function of the study group are shown in Table 2.

**Table 2:** Associations between chronic medication and erectile function ED: erectile dysfunction, ACEI: angiotensin-converting enzyme inhibitor, CCB: calcium channel blocker, HCTZ: hydrochlorothiazide, PPIs: proton pump inhibitors, PVD: peripheral vascular disease

Medication		Overall	ED patients	Non-ED patients	Pearson chi^2^	p-value
		N=170 (100%)	N=84 (49.4%)	N=86 (50.6%)		
Beta-blockers	Yes	36 (21.2%)	20 (23.8%)	16 (18.6%)	0.690	0.406
	No	134 (78.8%)	64 (76.2%)	70 (81.4%)		
ACEI	Yes	31 (18.2%)	14 (16.7%)	17 (19.8%)	0.274	0.601
	No	139 (81.8%)	70 (83.3%)	69 (80.2%)		
CCB	Yes	122 (71.8%)	62 (73.8%)	60 (69.8%)	0.343	0.558
	No	48 (28.2%)	22 (26.2%)	26 (30.2%)		
Allopurinol	Yes	58 (34.1%)	32 (38.1%)	26 (30.2%)	1.169	0.280
	No	112 (65.9%)	52 (61.9%)	60 (69.8%)		
Alpha-2 adrenergic agonists	Yes	37 (21.8%)	20 (23.8%)	17 (19.8%)	0.408	0.523
	No	133 (78.2%)	64 (76.2%)	69 (80.2%)		
Alpha-blockers	Yes	11 (6.5%)	9 (10.7%)	2 (2.3%)	4.941	0.026
	No	159 (93.5%)	75 (89.3%)	84 (97.7%)		
Anti-ischemic drugs	Yes	3 (1.8%)	2 (2.4%)	1 (1.2%)	0.364	0.618
	No	167 (98.2%)	82 (97.6%)	85 (98.8%)		
Psychotropic drugs	Yes	3 (1.8%)	1 (1.2%)	2 (2.3%)	0.316	1.000
	No	167 (98.2%)	83 (98.8%)	84 (97.7%)		
Antiarrhythmic drugs	Yes	2 (1.2%)	0 (0%)	2 (2.3%)	1.977	0.497
	No	168 (98.8%)	84 (100%)	84 (97.7%)		
Diuretics	Yes	24 (14.1%)	16 (19%)	8 (9.3%)	3.329	0.068
	No	146 (85.9%)	68 (81%)	78 (90.7%)		
Nitrates	Yes	1 (0.6%)	0 (0%)	1 (1.2%)	0.983	1.000
	No	169 (99.4%)	84 (100%)	85 (98.8%)		
H2 receptor agonists	Yes	2 (1.2%)	1 (1.2%)	1 (1.2%)	0.000	1.000
	No	168 (98.8%)	83 (98.8%)	85 (98.8%)		
PPIs	Yes	29 (17.1%)	12 (14.3%)	17 (19.8%)	0.902	0.342
	No	141 (82.9%)	72 (85.7%)	69 (80.2%)		
Statins	Yes	46 (27.1%)	24 (28.6%)	22 (25.6%)	0.192	0.661
	No	124 (72.9%)	60 (71.4%)	64 (74,4)		
Antidiabetic drugs	Yes	7 (4.1%)	4 (4.8%)	3 (3.5%)	0.175	0.718
	No	163 (95.9%)	80 (95.2%)	83 (96.5%)		
Aspirin (75 mg)	Yes	15 (8.8%)	12 (14.3%)	3 (3.5%)	6.158	0.013
	No	155 (91.2%)	72 (85.7%)	83 (96.5%)		
Anticoagulant drugs	Yes	6 (3.5%)	4 (4.8%)	2 (2.3%)	0.741	0.441
	No	164 (96.5%)	80 (95.2%)	84 (97.7%)		
Pentoxifylline for PVD	Yes	27 (15.9%)	16 (19%)	11 (12.8%)	1.245	0.264
	No	143 (84.1%)	68 (81%)	75 (87.2%)		
Immunosuppressive therapy	Tacrolimus	114 (67.1%)	58 (69%)	56 (65.1%)	0.300	0.861
	Cyclosporine	56 (32.9%)	26 (31%)	30 (34.9%)		

The laboratory characteristics at the first evaluation following hospital discharge and at 12 months after renal transplant are shown in Table 3. These laboratory tests did not show any statistically significant associations with the existence of ED.

**Table 3:** Laboratory characteristics in renal transplant recipients with and without erectile dysfunction at the first evaluation following hospital discharge and at 12 months after renal transplant +: Mann-Whitney test, ++: Student’ t-test *First evaluation following hospital discharge after renal transplant **Evaluation at 12 months after renal transplant RT: renal transplant, SD: standard deviation, ED: erectile dysfunction

First evaluation after RT* (mean±SD)	Total (N=170)	With ED (n=84)	Without ED (n=86)	p-value
Urea (mg/dL)	76.504±36.324	79.659±35.664	73.389±36.925	0.088^+^
Creatinine (mg/dL)	1.545±0.591	1.561±0.562	1.528±0.623	0.272^+^
Hemoglobin (g/dL)	11.309±1.756	11.198±1.592	11.417±1.905	0.434^++^
Hematocrit (%)	34.402±5.141	34.251±4.948	34.551±5.352	0.717^++^
Cholesterol (mg/dL)	220.49±48.144	222.68±44.505	218.33±51.685	0.576^++^
Triglycerides (mg/dL)	201.44±96.611	198.75±82.722	204.09±109.092	0.756^+^
Uric acid (mg/dL)	5.969±1.652	6.141±1.565	5.800±1.729	0.229^++^
Values 12 months after RT ** (mean±SD)				
Urea (mg/dL)	51.804±19.539	51.987±19.872	51.622±19.326	0.833^+^
Creatinine (mg/dL)	1.503±1.057	1.385±0.402	1.622±1.439	0.196^+^
Hemoglobin (g/dL)	14.071±1.952	13.787±2.007	14.355±1.865	0.068^++^
Hematocrit (%)	42.902±6.958	42.694±7.014	43.113±6.940	0.646^+^
Cholesterol (mg/dL)	201.41±44.463	203.76±39.601	199.13±48.890	0.440^+^
Triglycerides (mg/dL)	169.02±74.780	174.84±73.679	163.35±75.881	0.226^+^
Uric acid (mg/dL)	6.744±1.532	6.750±1.657	6.739±1.407	0.966^++^

The laboratory tests and analyses performed at the time of the study’s inclusion are shown in Table 4. Once more, no statistically significant associations were seen between those with and without erectile dysfunction.

**Table 4:** Laboratory characteristics of kidney transplant recipients with and without erectile dysfunction at the time of the study’s inclusion +: Mann-Whitney test, ++: Student’s t-test SD: standard deviation, ED: erectile dysfunction

Actual values (mean±SD)	Total (N=170)	With ED (n=84)	Without ED (n=86)	p-value
Urea (mg/dL)	51.641±21.728	52.012±21.447	51.274±22.124	0.635^+^
Creatinine (mg/dL)	1.545±0.960	1.472±0.571	1.617±1.230	0.512^+^
Hemoglobin (g/dL)	14.062±1.842	13.896±1.986	14.225±1.683	0.248^++^
Hematocrit (%)	41.786±5.284	41.126±5.701	42.438±4.780	0.107^++^
Cholesterol (mg/dL)	198.84±47.007	188.53±37.308	208.79±53.110	0.005^++^
Triglycerides (mg/dL)	166.49±87.402	154.58±72.061	177.96±99.084	0.093^+^
Uric acid (mg/dL)	6.693±1.431	6.745±1.342	6.638±1.525	0.652^++^

## Discussion

The high prevalence of erectile dysfunction among patients with chronic renal failure has long been recognized for years. Research from the 1970s showed how crucial it was to improve quality of life through transplant and recover one’s normal level of erectile function [9,10]. Salvatierra et al. [10] conducted a study in which 130 young males (aged 30-60) completed a questionnaire on their sexual satisfaction before and after undergoing a kidney transplant. According to his findings, potency and libido significantly changed after transplantation without any evidence of a significant relationship with age, the etiology of chronic renal disease, the kind of donor, concomitant drugs, or creatinine level.

It is debatable and possibly multifactorial what induces sexual dysfunction in renal transplant patients. Some of the factors that have been recognized as related to persistent ED include age, anxiety, drug side effects, interference with penile vascularization, failure to adjust hormonal imbalances, or an underlying pathological process [11].

Before starting hemodialysis, patients with chronic kidney disease had a high prevalence of sexual dysfunction (90%), and it remained elevated (60%-70%) during the hemodialysis period, with a range of clinical manifestations, from a lack of sexual desire to the inability to maintain an erection, from reduced sex hormone levels to infertility, from low self-esteem to depressive episodes [12]. In a prospective research by Nassir et al. [3], 52 patients’ erectile dysfunction was examined both before and after kidney transplantation. The age of patients undergoing renal transplantation is decreasing due to easier access to the procedure, so erectile function among the male population is of higher importance. The study’s findings showed that while dialysis did not enhance erectile function, those who had kidney transplantation saw a substantial improvement in sexual function.

Although higher than that observed in Spain [13], in the study by Rosen et al. [14], the overall prevalence for all types of chronic kidney disease (43% of patients on hemodialysis, 25% of patients on peritoneal dialysis, and 21% of renal recipients) was similar to the rate observed in the general population in the USA [15] for the same age group. Burgos et al. [9] reported in a prospective trial that sexual dysfunction reduced from 60% to 45% following renal transplant; this is a different rate from that observed in the present investigation for transplant recipients. Comparable to our analysis and with similar average age, Peşkircioğlu et al. [16] observed an ED prevalence of 32.3% (42.5 versus 45.4 in our study).

Age is a widely accepted risk factor for sexual dysfunction, and many studies have demonstrated the importance of age on the occurrence of ED among transplant patients [6,17,18]. In the study by Malavaud et al. [17] of 271 post-renal transplant recipients, the mean age of adult males with sexual dysfunction was 49.2 years, compared to 44.5 years for patients without ED, which is comparable to our findings. According to investigations by El-Bahnasawy et al. [6], patients with ED were older on average than those without sexual dysfunction (43.6 years compared to 34.9 years, respectively). Mirone et al. [5] found that in people under the age of 45, renal transplant had no beneficial effects on erectile dysfunction and sometimes was even reported to worsen by the recipients.

There are several well-known sexual dysfunction causes, including high blood pressure, diabetes mellitus, hyperlipidemia, and uremic toxins. Despite potential benefits following renal transplantation, endothelial dysfunction persists [19]. These findings should make clinicians aware of the relevance of sexual dysfunction among renal recipients because it may be an indicator of cardiovascular risk considering that major cardiovascular events are the main noninfectious cause of death in this group of recipients [20]. Diabetes mellitus is another common cause of erectile dysfunction, and it is considered that erectile dysfunction results from the accelerated development of angiopathy and neuropathy. Al Khallaf [21] observed that both nondiabetic males with kidney transplants and nondiabetic patients receiving dialysis had a poor erectile function and intercourse pleasure but normal orgasmic function. It was concluded that uremic patients’ erectile function is not fully normalized by either hemodialysis or kidney transplantation. Although a statistically significant correlation could not be established between the degree of dysfunction and the frequency of comorbidities in our study, we have observed that patients with erectile dysfunction have multiple associated diseases such as peripheral vascular and central heart disease, metabolic syndrome, and endocrine dysfunctions.

As calcineurin inhibitors such as tacrolimus and cyclosporine, which are gonadotoxic medications, constitute the basis for immunosuppression in transplant patients, additional research explores a variety of immunosuppressive treatment regimens that may have a detrimental impact on sexual function [22,23]. Although cyclosporine is known to impair endothelial function [24], the investigation of Bellinghieri et al. [25] into the impact of cyclosporine therapy on sexual hormone profiles revealed no connection between testicular function and the hormone profiles. Tacrolimus therapy was linked to regular erectile function, according to the research by Rebollo et al. [18]. No difference in the frequency of sexual dysfunction was found in our study, which included patients taking tacrolimus and cyclosporine (66.5% and 33.5%, respectively). However, our research did identify a certain correlation between erectile dysfunction and both alpha-blockers and aspirin (75 mg).

Hemoglobin level is one factor that affects erectile dysfunction in renal transplant recipients, and patients who are anemic have two times higher risk of getting ED than those who are not anemic [6]. While serum hemoglobin has a direct relationship with the presence of ED among hemodialyzed patients, serum urea or creatinine levels had a substantial negative association with the presence of ED among dialyzed patients [26]. We collected data on levels of urea and creatinine, hemoglobin, and lipid metabolism immediately following surgery, at six months, at 12 months, and again at the moment of study inclusion. However, when comparing the group of individuals with sexual dysfunction to those with normal erectile function, our research was unable to establish a statistically significant correlation.

There are some limitations to our study. First, patients did not complete the IIEF questionnaire at least six months after the kidney transplant but rather at various intervals thereafter. The study’s observational design, lack of hormonal characteristics that could affect sexual function, absence of clinical manifestations related to the severity of the disease, lack of a control group, and the fact that it was conducted in a single medical facility are additional flaws that should be taken into consideration.

In our view, this group of patients is growing annually, appears to be younger, and requires counseling and, where appropriate, therapy. Additional research is required to understand the incidence, risk factors, and severity of erectile dysfunction among those who underwent a kidney transplant.

## Conclusions

Due to its incidence and risk factors, sexual dysfunction is a significant problem for kidney transplant patients, and its frequency rises with age. The etiology is complex and may be due to a combination of variables; however, the two medical conditions may share many risk factors. Although most of the patients were young, only a small percentage of the study group had a normal sexual function. Among the several medications that this group of patients needed to take, alpha-blockers and aspirin (75 mg) are associated with sexual dysfunction, but these findings need to be validated in a prospective trial. Further research is needed to fully understand the complex interplay of these factors in the development of these disorders.

## References

[1] Webster AC, Nagler EV, Morton RL, Masson P (2017). Chronic kidney disease. Lancet.

[2] McKercher C, Sanderson K, Jose MD (2013). Psychosocial factors in people with chronic kidney disease prior to renal replacement therapy. Nephrology (Carlton).

[3] Nassir A (2009). Sexual function in male patients undergoing treatment for renal failure: a prospective view. J Sex Med.

[4] Mehrsai A, Mousavi S, Nikoobakht M, Khanlarpoor T, Shekarpour L, Pourmand G (2006). Improvement of erectile dysfunction after kidney transplantation: the role of the associated factors. Urol J.

[5] Mirone V, Longo N, Fusco F, Verze P, Creta M, Parazzini F, Imbimbo C (2009). Renal transplantation does not improve erectile function in hemodialysed patients. Eur Urol.

[6] El-Bahnasawy MS, El-Assmy A, El-Sawy E, Ali-El Dein B, Shehab El-Dein AB, Refaie A, El-Hammady S (2004). Critical evaluation of the factors influencing erectile function after renal transplantation. Int J Impot Res.

[7] Rosen RC, Riley A, Wagner G, Osterloh IH, Kirkpatrick J, Mishra A (1997). The international index of erectile function (IIEF): a multidimensional scale for assessment of erectile dysfunction. Urology.

[8] Cappelleri JC, Rosen RC, Smith MD, Mishra A, Osterloh IH (1999). Diagnostic evaluation of the erectile function domain of the International Index of Erectile Function. Urology.

[9] Burgos F, Pascual J, Gomez V, Orofino L, Liaño L, Ortuño J (1997). Effect of kidney transplantation and cyclosporine treatment on male sexual performance and hormonal profile: a prospective study. Transplant Proc.

[10] Salvatierra O Jr, Fortmann JL, Belzer FO (1975). Sexual function of males before and after renal transplantation. Urology.

[11] El-Bahnasawy MS, El-Assmy A, Dawood A, Abobieh E, Dein BA, El-Din AB, El-Hamady Sel-D (2004). Effect of the use of internal iliac artery for renal transplantation on penile vascularity and erectile function: a prospective study. J Urol.

[12] Toorians AW, Janssen E, Laan E (1997). Chronic renal failure and sexual functioning: clinical status versus objectively assessed sexual response. Nephrol Dial Transplant.

[13] Martin-Morales A, Sanchez-Cruz JJ, Saenz de Tejada I, Rodriguez-Vela L, Jimenez-Cruz JF, Burgos-Rodriguez R (2001). Prevalence and independent risk factors for erectile dysfunction in Spain: results of the Epidemiologia de la Disfuncion Erectil Masculina Study. J Urol.

[14] Rosen RC, Cappelleri JC, Smith MD, Lipsky J, Peña BM (1999). Development and evaluation of an abridged, 5-item version of the International Index of Erectile Function (IIEF-5) as a diagnostic tool for erectile dysfunction. Int J Impot Res.

[15] Feldman HA, Goldstein I, Hatzichristou DG, Krane RJ, McKinlay JB (1994). Impotence and its medical and psychosocial correlates: results of the Massachusetts Male Aging Study. J Urol.

[16] Peşkircioğlu L, Tekin MI, Demirağ A, Karakayali H, Ozkardeş H (1998). Evaluation of erectile function in renal transplant recipients. Transplant Proc.

[17] Malavaud B, Rostaing L, Rischmann P, Sarramon JP, Durand D (2000). High prevalence of erectile dysfunction after renal transplantation. Transplantation.

[18] Rebollo P, Ortega F, Valdés C, Fernández-Vega F, Ortega T, García-Mendoza M, Gómez E (2003). Factors associated with erectile dysfunction in male kidney transplant recipients. Int J Impot Res.

[19] Mark PB, Murphy K, Mohammed AS, Morris ST, Jardine AG (2005). Endothelial dysfunction in renal transplant recipients. Transplant Proc.

[20] Nieto T, Inston N, Cockwell P (2016). Renal transplantation in adults. BMJ.

[21] Al Khallaf HH (2010). Analysis of sexual functions in male nondiabetic hemodialysis patients and renal transplant recipients. Transpl Int.

[22] Hošková L, Málek I, Kopkan L, Kautzner J (2017). Pathophysiological mechanisms of calcineurin inhibitor-induced nephrotoxicity and arterial hypertension. Physiol Res.

[23] Lessan-Pezeshki M, Ghazizadeh S (2008). Sexual and reproductive function in end-stage renal disease and effect of kidney transplantation. Asian J Androl.

[24] Nickel T, Schlichting CL, Weis M (2006). Drugs modulating endothelial function after transplantation. Transplantation.

[25] Bellinghieri G, Santoro D, Lo Forti B, Mallamace A, De Santo RM, Savica V (2001). Erectile dysfunction in uremic dialysis patients: diagnostic evaluation in the sildenafil era. Am J Kidney Dis.

[26] Ali ME, Abdel-Hafez HZ, Mahran AM (2005). Erectile dysfunction in chronic renal failure patients undergoing hemodialysis in Egypt. Int J Impot Res.

